# Changes of 25-OH-Vitamin D during Overwintering at the German Antarctic Stations Neumayer II and III

**DOI:** 10.1371/journal.pone.0144130

**Published:** 2015-12-07

**Authors:** Mathias Steinach, Eberhard Kohlberg, Martina Anna Maggioni, Stefan Mendt, Oliver Opatz, Alexander Stahn, Josefine Tiedemann, Hanns-Christian Gunga

**Affiliations:** 1 Center for Space Medicine and Extreme Environments Berlin, Institute for Physiology, Charité Universitätsmedizin Berlin, Berlin, Germany; 2 Alfred Wegener Institute for Polar and Marine Research, Bremerhaven, Germany; 3 Department of Biomedical Sciences for Health, Università degli Studi di Milano, Milan, Italy; University of Alabama at Birmingham, UNITED STATES

## Abstract

**Purpose:**

Humans in Antarctica face different environmental challenges, such as low ultra-violet radiation, which is crucial for vitamin D production in humans. Therefore we assessed changes in 25-OH-vitamin D serum concentration during 13 months of overwintering at the German Stations Neumayer II and III (2007–2012). We hypothesized that (i) 25-OH-vitamin D serum concentration would significantly decrease, (ii) changes would be affected by age, gender, baseline (i.e. pre-overwintering) fat mass, baseline 25-OH-vitamin D serum concentration, and station residence, and (iii) our results would not differ from similar previous studies in comparable high latitudes.

**Materials & Methods:**

25-OH-vitamin D serum concentrations were determined before, after, and monthly during the campaigns from venous blood samples of n = 43 participants (28 men, 15 women). Baseline fat mass was determined via bio impedance analysis and body plethysmography. Data were analyzed for change over time, dependency on independent parameters, and after categorization for sufficiency (>50nmol/l), insufficiency (25-50nmol/l), and deficiency (<25nmol/l). Results were compared with data from similar previous studies.

**Results:**

We found a significant decrease of 25-OH-vitamin D with dependency on month. Age, gender, fat mass, and station residence had no influence. Only baseline 25-OH-vitamin D serum concentrations significantly affected subsequent 25-OH-vitamin D values.

**Conclusions:**

Overwinterings at the Antarctic German research stations Neumayer II and III are associated with a decrease in 25-OH-vitamin D serum concentrations, unaffected by age, gender, baseline fat mass, and station residence. Higher baseline vitamin D serum concentrations might protect from subsequent deficiencies. Residence at the Neumayer Stations may lead to lower vitamin D serum concentrations than found in other comparable high latitudes.

## Introduction

Life and work of humans in high latitudes, i.e. latitudes close to polar regions, such as the Arctic or Antarctic, are often associated with adverse conditions such as very cold climate, changed circadian cycle, and altered exposure to ultra-violet (UV)-light [1–3]. In addition, extended human stays in Antarctic research stations may be associated with psychosocial isolation, sensory deprivation, and exhaustion [4,5]–a combination of adverse factors, which subsequently may lead to challenges to hormonal, metabolic, and immune functions [6–8]. Polar regions, such as the Antarctic, receive less intensive solar radiation because the sunlight hits the Earth at an oblique angle. In addition, the Antarctic climate is dominated by seasonal changes. Depending on the latitude, months of complete darkness during the Antarctic winter alternate with months of 24-hrs bright daylight in the Antarctic summer [9]. This has particular consequences on vitamin D homeostasis for humans residing there [10–12]. Germany currently operates the year-long inhabited research station Neumayer III, which was inaugurated in February 2009; it replaced the previous station Neumayer II, which operated from 1992 to 2009, and which had to be abandoned because its structural integrity could no longer be maintained, as it was located underground within ice [13]. Both stations served as the location of the presented study located at 70° 40’ S, 08° 16‘ W.

The collective term "vitamin D" (calciferol) combines vitamin D_3_ (cholecalciferol) and vitamin D_2_ (ergocalciferol). The formation of vitamin D in the human skin–depending on sunlight exposure altered by geographical location, altitude, season, clothing, occupation, age and ethnicity–makes up to 95% of the vitamin D requirement, indicating the importance of adequate UV-light for vitamin D formation [14–16]. A photochemical conversion of the pro-vitamin D_3_ (7-dehydrocholesterol 7-DHC) by UV-light of wavelengths 290–315 nm causes the formation of pre-vitamin D_3_, which is converted to vitamin D_3_ through thermal isomerization. A small portion of vitamin D_3_ is stored in adipose tissue and skeletal muscle [17] from where it can be released in times of deficiency resulting in a half-life of up to two months [15]. In the liver and kidneys the final activation steps to 1,25-dihydroxyvitamin D (calcitriol) are catalyzed. 1α-hydroxylase activity has been found in several other tissues suggesting the ability to produce 1,25-dihydroxyvitamin D outside the kidneys [18,19]. Its target organs are the intestine, bone, kidney, adrenal gland and others [14,20]. 1,25-dihydroxyvitamin D has calcemic and non-calcemic effects. The former are to maintain the calcium and phosphate homeostasis through regulation of intestinal and renal calcium absorption, bone tissue calcification, and inhibition of parathyroid hormone [21]. The latter serve to regulate cell growth and differentiation, to regulate immune function, to control the renin-angiotensin system, to control muscular function, brain development and mood [22–25]. Other positive effects of vitamin D could be shown on the function of the nervous system [26], inhibition of diseases like the metabolic syndrome, susceptibility to infection and several types of cancer [27–29]. Supplementation with vitamin D has been shown to reduce overall mortality [30], while genetically low vitamin D serum concentrations seem to be accociated with increased all-cause mortality [31]. 25-OH-vitamin D serum concentration has been accepted and used as an accurate measure of a humans’ vitamin D content, which considers both intake from diet and skin production [32–34]. The literature offers a broad range of 25-OH-vitamin D values that are considered above or below certain thresholds for optimal health conditions. Serum concentrations of 25-OH-vitamin D of at least 75 nmol/l have been shown to effectively prevent fractures [35] and are seen by some authors as the lower limit to maintain health [36]. Other authors have argued that concentrations below 80 nmol/l may already be deficient [37] and concentrations above 100 nmol/l are to be considered optimal [38]. Lower more conservative thresholds of at least 50 nmol/l have been introduced as being sufficient [39,40]. Around one billion people worldwide are estimated to be vitamin D deficient [41]. Wearing cold protective clothing, shielding the skin for cultural or religious reasons, residence at high latitudes, and having dark skin pigmentation are known to increase the risk for vitamin D deficiency [20,41,42]. Especially at higher latitudes, during local wintertime, vitamin D production requires longer exposure time [43] or ceases completely [44]. This decrease has significant consequences for people inhabiting these areas either as indigenous population [33,45] or as researchers based in Antarctic stations [46]. Although it was shown that locally produced foods at high latitudes like fatty fish and wild game can be sufficient to maintain adequate vitamin D serum concentrations [47,48], it has been argued that this traditional diet might gradually be replaced by westernized foods [49], which might not be sufficient to provide enough vitamin D at higher latitudes [50]. Notably, foods consumed at the Neumayer Stations were scarce with regard to fresh vegetables and fruits, they consisted mostly of processed foods, which are considered to be low in vitamin D content [16,51], thus possibly increasing the risk for vitamin D deficiencies.

The aim of the study was therefore to assess changes in 25-OH-vitamin D serum concentration in overwinterers of the German research stations Neumayer II and III for 13 months during a total of six campaigns from 2007 to 2012. We hypothesized that vitamin D would be significantly decreased during the Antarctic winter. Furthermore, we assessed whether these changes were affected by age, gender, baseline fat mass, baseline 25-OH-vitamin D serum concentration, and the type of inhabited station (station II located below ground, station III above ground). In addition, we compared our findings with results from previous studies of similar latitudes and measurement periods.

## Materials and Methods

### General circumstances

The crew of the Neumayer Stations II and III in the Antarctic winter consisted of employees from different fields and professions (meteorologists, chemists, geophysicists, electricians, engineers, computer technicians, a cook, and a medical doctor who also acted as commander of the crew). The members of each overwintering crew resided at the Antarctic station for 13 to 14 months (from November prior, to January past the respective overwintering year). The recruitment and training of the crews was carried out by the “Alfred Wegener Institute for Polar and Marine Research” [13]. Aside from their respective fields of occupation, all crewmembers had to attend duties related to station maintenance. Due to the risks involved (injuries due to fall, frostbite and hypothermia), the outside-activity during the Antarctic winter was reduced to a minimum; however, each crewmember was equipped with cold-protection clothing and emergency equipment [13]. The supply of food to the Neumayer Stations II and III was limited to accessibility time periods. The food was composed primarily of frozen products. Fresh fruit and vegetables were only available for a short period during summer season in November until February. A rationing was not performed, and no restrictions of caloric intake were applied [13]. For the purpose of this study, six overwintering seasons from 2007 to 2012 were investigated. Per overwintering season, nine adult crewmembers of Caucasian descent, lived and worked at the station. The recruitment process of the “Alfred Wegener Institute” included a medical and psychological screening and ensured that crew members were of good physical and mental health and were not taking any medication (except for oral contraceptives by the female participants). During their recruitment, they were invited to participate in this prospective study; intake of vitamin D supplements was the only exclusion criterion. One person each in 2008 and 2010 decided not to partake as did two individuals in 2009 and 2011 and four in 2012. One subject of the overwintering crew in 2012 took vitamin D supplements and was therefore excluded from the study. Thus, from a total of 54 participants of the six overwintering seasons a total of 43 (28 men and 15 women) took part in the study. After comprehensive explanations of study details and implications, the participants were given due time to clarify further questions and to express their desire to partake in the study. All of them gave their written informed consent. The study was approved by the local Ethics Committee at Charité Universitätsmedizin Berlin. All procedures were conducted in accordance with the Declaration of Helsinki regarding human subjects.

### German Antarctic Stations Neumayer II and III

**Fig 1:**
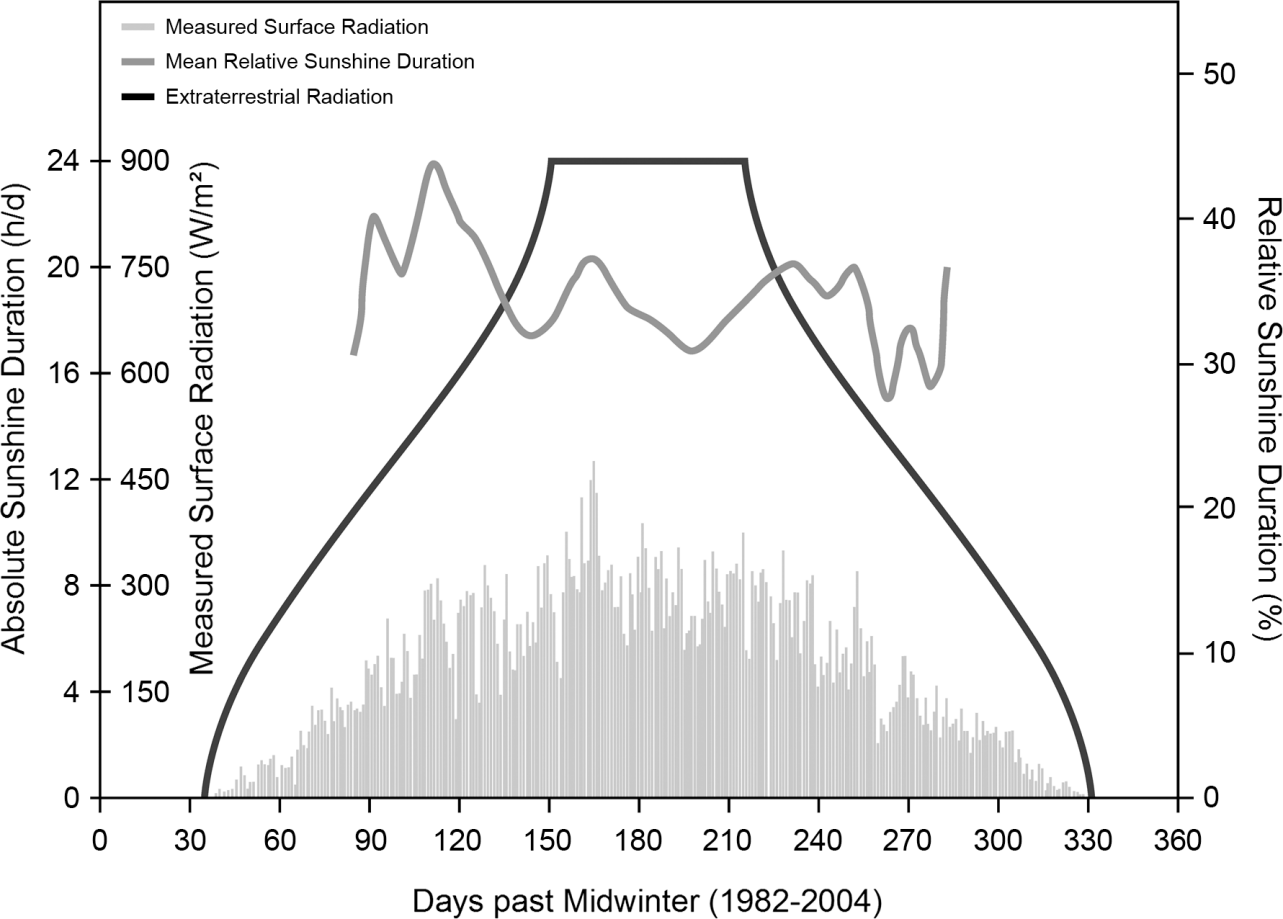
Duration of sunshine at the Neumayer Stations in Antarctica (1982–2004). Plots of extraterrestrial radiation (black), relative sunshine duration (dark grey) and measured surface radiation (light grey) at the location of the Neumayer Stations. The x-axis displays the time in days with “0” representing midwinter (June 21st). For a period of about 100 to 120 days around midwinter virtually no light reached the surface. (Unpublished personal communication in 2012 by G. König-Langlo, “Alfred Wegener Institute for Polar and Marine Research”, Bremerhaven).

German Antarctic research stations Neumayer Station II and Neumayer Station III, operated by the “Alfred Wegener Institute for Polar and Marine Research” (Bremerhaven, Germany), were the locations of the investigation. Station II was and station III is located in Atka-bay 70° 40’ S, 8° 16’ W in the Ekström-shelf ice [13]. Adverse weather conditions during the darkness phase made it nearly impossible to reach the station by airplane or snow mobiles, which led to complete isolation of the inhabitants; merely an Internet connection and a satellite phone enabled contact with the outside world. Emergency rescues would have been practically impossible. Supplies were transported to the station once a year by the research vessel "Polarstern" [13]. The location of the research stations at 70° south determined the amount of sunshine that reached the surface [9]. As indicated in Fig 1, for a period of about 60 days around midwinter (21st of June), virtually no sunlight reached locations at that latitude, while for another 30 days before and after that period the sunshine radiation was very low, which led to periods of complete darkness between the end of April and the end of August. Less than 50 W/m^2^ were measured between beginning of April and the end of September, and less than 5 W/m^2^ from the mid of May until the beginning of August, respectively. Sunshine duration (with the respective low energies) was less than about 5 hrs/d from mid-April to the end of August and virtually zero from the mid-May to the beginning of August. Sunshine from November to January during the Antarctic summer, however, can reach up to 24 hrs with 300 to 450 W/m^2^ (unpublished personal communication in 2012 by G. König-Langlo, “Alfred Wegener Institute for Polar and Marine Research”, Bremerhaven). This also led to very low temperatures. At 12 p.m. the mean ambient temperature at the Neumayer Stations between 2008–2011 ranged around -2.7 ± 2.0°C (n = 124) and -25.2 ± 7.2°C (n = 124) in January and July, respectively [13].

Stations Neumayer II and III serve to gather data in aerial-chemical, geophysical and meteorological investigations, and, since the beginning of 2000, also for medical and physiological studies. Station Neumayer II was located underground and consisted of a tube construction (90-m in length and about 8 m in diameter) connected to each other. Air-conditioned ship containers served as the laboratories, the kitchen, the workshops, the sickbay as well as the radio room and the thaw room. In a third tube chill camps, waste disposal and garbage storage as well as the vehicle garage were accommodated [13]. Since February 2009, the new Neumayer Station III has been in operation. This is the first German Antarctic station that combines human stay and research on a platform above the ice surface with a garage built within ice. This station has the unique quality to lift itself up according to snow accumulation by the use of hydraulic technics attached to the supporting feet [13]. This station also offers air-conditioned laboratories and accommodation areas as well as a sauna, a dining room, a conference room, medical treatment rooms, and storage and technical rooms.

### Anthropometric data of the subjects

Anthropometric data of the study participants were gathered using standard equipment (medical scale and height meter, SECA, Germany) in minimal clothes. Baseline fat mass was determined using the bioelectrical impedance analysis (BIA 101, AKERN, Italy) according to Sun et al. 2003 [52] for campaigns 2007–2011 and the Whole Body Plethysmography (BODPOD, COSMED, USA) for campaign 2012. Both are established methods for determining body composition [53–55] while the BIA is gaining increasing interest in field conditions due to its mobility [56].

### Blood samples and biochemical analysis

Venous blood samples were collected in the morning before breakfast and after an overnight fast as part of the crew’s routine medical check-ups by the stations’ physician using SARSTEDT Monovettes for blood collection (SARSTEDT, Nümbrecht, Germany). The amount and frequency of blood collection varied between overwintering seasons, due to different operational constraints. Samples were assigned to their nearest two-week time slot (see Table 1) for the purpose of analysis. After collection, whole blood was immediately centrifuged (15 min at 2500 rpm at room temperature), the serum aliquoted, stored at -20°C, and transported observing the cooling chain to Germany for analysis using the ELISA-method (IDS, Frankfurt/Main, Germany (Ref AC57F1)), standard values 47.7–144 nmol/l (male and female, 5^th^ to 95^th^ percentile). The intra- and inter-assay coefficient of variation (CV) ranged between 4.6% at 40.3 nmol/l and 8.7% at 132 nmol/l.

**Table 1:** Times of measurement and number of subjects.

		Times of Measurement																									
Season	n	Jan		Feb		Mar		Apr		May		Jun		Jul		Aug		Sep		Oct		Nov		Dec		Jan^†^	
2007	9	•					•					•				•					•					•	
2008	8	•				•				•				•				•				•					
2009	7	•					•					•				•				•				•			
2010	8					•				•				•					•				•				•
2011	7	•				•				•		•		•		•		•				•		•			
2012	4					•		•		•		•		•		•		•		•		•					
n	43	31		0		43		4		27		27		27		27		27		20		27		14		17	

Number of participants and respective times of measurement per overwintering season and month of measurement on a two-week basis

^†^ denotes the January at the end of the overwintering

### Statistics

Descriptive data are reported as means and standard deviations (median and 25th and 75th percentile respectively for fat mass). Data for 25-OH-vitamin D serum concentrations are graphically displayed as boxplots (25th to 75th percentile (box), median (line), values within 1.5 times interquartile range (whisker) and outlier values outside 1.5 times interquartile range (circles)). Differences over time were assessed by one-way repeated measures ANOVA for all subjects, as well as separately for the two sexes. Holm-Šidák corrected post-hoc multiple comparison tests were conducted to track significant main effects. Results were plotted as a scatterplot where measurement results were combined to represent one period of thirteen months on a two-week basis according to when measurements took place. A quadratic curve was fitted to describe the change over time. In addition, a regression analysis was performed for the dependency of 25-OH-vitamin D serum concentrations on local daily sunlight-radiation at noon averaged per two-week intervals measured in W/m^2^ after compensation of the phase difference between these two parameters. For the purposes of categorizing vitamin D insufficiency, rather conservative threshold values of > 50 nmol/l as sufficient, 25–50 nmol/l as insufficient and < 25 nmol/l as deficient were used [39,40]. The percentage for each category was calculated per month for the respective number of participants for both sexes, as well as for men and women separately. Of special interest was the period of complete darkness. This period was i) defined to be from August to September in accordance with the amount of local sunshine radiation (see Fig 1) and ii) to include the effect of half-life of 25-OH-vitamin D of up to three weeks [57]. Regarding the change from the beginning of the overwintering to the period of complete darkness and towards the end of the overwintering period, the measured values of 25-OH-vitamin D were taken for each participant at the first measurement (either in January or March, n = 43), at August or September (for participants who had blood samples taken in both months a mean value was calculated from the two, n = 43) and at December or January (again for participants who had blood samples taken in both months a mean value was calculated from the two, n = 31). These values were plotted in a lineplot respective to the defined categories sufficient (Su: > 50 nmol/l), insufficient (In: 25–50 nmol/l), and deficient (De: < 25 nmol/l). Analysis of covariance (ANCOVA) and multiple linear correlation were performed for 25-OH-vitamin D serum concentration during August-September and December-January being the dependent variable and the parameters age (years), gender (male or female), fat mass (kg), 25-OH-vitamin D serum concentration at the first measurement (nmol/l), and inhabited Neumayer Station (II or III) being the covariates respectively. Fat mass was chosen over BMI as having a greater influence on metabolism and hormonal balance [58]. Using both fat mass and BMI was rejected in order to avoid multicollinearity. Finally, differences between our results and data from previous comparable studies were investigated. From the limited numbers of studies concerning changes in 25-OH-vitamin D at high latitudes, those were selected, that had previously published their results regarding 25-OH-vitamin D serum concentrations of subjects without vitamin D supplementation, at latitudes from 64 to 78 degrees latitude (north or south), comparable measurement period, and that had stated their results as means ±SD, number of participants, and regarding where and when the blood samples were taken: location, latitude and time period. These studies used the following methods to determine 25-OH-vitamin D serum concentrations: a competitive protein binding method [10] further described here [59], use of radioimmunoassay kits (DIASORIN, Stillwater, USA) [46,60,61], use of enzyme immunoassay kits (IDS, Bolden, UK) [62], use of a high-pressure liquid chromatography-atmospheric pressure chemical ionization-mass spectrometry (VITAS, Olso, Norway) [63], and use of a reverse-phase high-performance liquid chromatography system (UV6000; THERMOFINNIGAN, San Jose, USA) [64,65]. Regression analysis and one-way ANOVA were performed with those results and the results of this study, taken as means ±SD during August and September (n = 43); subsequent multiple comparison analysis via Holm-Šidák correction method was applied. All data were handled through MICROSOFT EXCEL Version 2007 (12.0.4518) and analyzed using SYSTAT SIGMAPLOT Version 12 (12.2.0.45) and 13 (13.0.0.83). A two-sided p-value of below 0.05 was considered to be an indicator for statistical significance.

## Results

The overwinterers’ anthropometric data at the beginning of their respective overwintering campaign are shown in Table 2. Mean BMI measured in seasons 2007 to 2011 decreased significantly from 25.0 kg/m^2^ ±3.3 to 24.9 kg/m^2^ ±3.1 during the end of August and further to 24.8 kg/m^2^ ±3.2 at the end of the campaigns (p<0.001), as did FM (median 25 and 75% quartile: 17.5 kg 12.5–20.8%, 16.1 kg 12.9–20.3%; 15.7 kg 13.3–19.6%), however, these changes in FM were not significant.

**Table 2:** Anthropometric parameters.

Parameter	Mean	±SD	Minimum	Maximum
Age (years)	36.5	9.1	25	60
Body Mass (kg)	77.5	13.1	55.9	109.0
Height (cm)	176.4	8.4	160.0	196.0
BMI (kg/m^2^)	24.8	3.3	19.2	34.2
Fat Mass (kg)	16.7	5.8	5.1	31.6
Fat Mass (%)	21.7	6.2	5.5	37.0

Anthropometric parameters of the study sample (n = 43, men = 28, women = 15)

**Fig 2:**
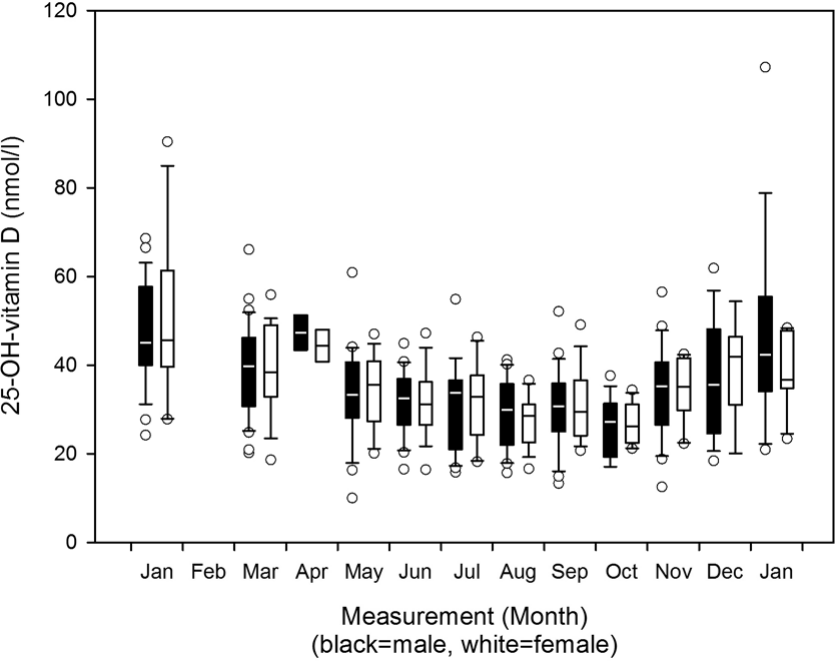
25-OH-vitamin D serum concentrations 2007–2012: Boxplots per month separate for the sexes. Changes of 25-OH-vitamin D serum concentrations from 2007 to 2012, boxplots per month separate for male and female participants (refer to Table 3 for n per month).

**Fig 3:**
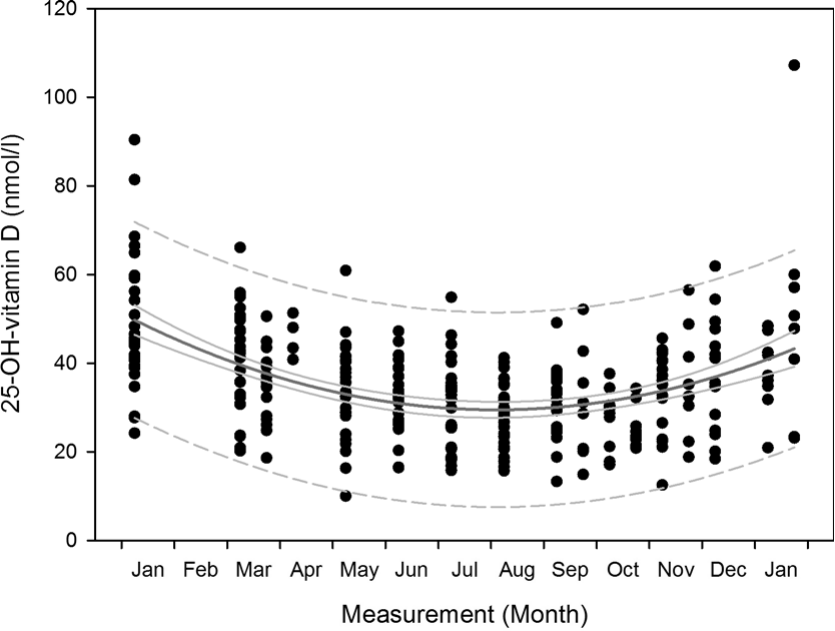
25-OH-vitamin D serum concentrations 2007–2012: Scatterplot per two-week measurement and nonlinear regression curve. Changes of 25-OH-vitamin D serum concentrations from 2007 to 2012, scatterplot per two-month measurement for all participants (refer to Table 3 for n per month) with nonlinear regression curve (dark grey curve), 95% confidence intervals (light grey curve) and 95% prediction intervals (dashed grey curve).

We observed a decrease in 25-OH-vitamin D mean serum concentrations towards the months of complete darkness and a subsequent increase during the post darkness period in men and women as Fig 2 shows. One-way RM ANOVA yielded significant results for all subjects (p<0.001), as well as for both male and female subjects separately (p<0.001). The results of the subsequent all pairwise multiple comparison for all subjects and for male and female subjects separately are displayed in Table 3. There were no differences between the sexes (p = 0.823). The scatterplot regarding periodicity of consecutive years is shown in Fig 3. The fitted quadratic regression curve follows the Eq (a):

$$y=0.108x^{2}-3.17x+52.81,\;r=0.490,\;r^{2}=0.240.$$ (a)

**Table 3:** 25-OH-vitamin D 2007–2012: mean values ±SD, n and all pairwise multiple comparison procedures between months for all participants and separated for the sexes.

										p-values						
Gender	n	25-OH-vitamin D		Month	Jan	Mar	Apr	May	Jun	Jul	Aug	Sep	Oct	Nov	Dec	Jan^†^
		mean	±SD													
All	31	48.4	15.2			<0.001	-	<0.001	<0.001	<0.001	<0.001	<0.001	<0.001	<0.001	<0.001	0.049
Male	20	47.0	12.1	Jan		<0.001	-	<0.001	<0.001	<0.001	<0.001	<0.001	<0.001	<0.001	0.038	-
Female	11	51.0	20.2			<0.001	-	<0.001	<0.001	<0.001	<0.001	<0.001	<0.001	<0.001	-	0.005
All	43	39.0	11.0				-	0.001	0.002	<0.001	<0.001	<0.001	<0.001	0.004	-	-
Male	28	39.0	11.0	Mar			-	0.008	-	<0.001	0.004	<0.001	0.019	0.023	-	-
Female	15	38.8	11.2				-	-	-	-	<0.001	-	0.009	-	-	-
All	4	45.9	4.7					-	-	-	0.044	0.045	-	-	-	-
Male	2	47.4	5.6	Apr				-	-	-	-	-	-	-	-	-
Female	2	44.4	5.1					-	-	-	-	-	-	-	-	-
All	27	33.8	10.6						-	-	-	-	-	-	0.045	<0.001
Male	18	33.8	11.6	May					-	-	-	-	-	-	-	<0.001
Female	9	33.7	9.0						-	-	-	-	-	-	-	-
All	27	31.6	7.7							-	-	-	-	-	-	<0.001
Male	16	31.6	7.5	Jun						-	-	-	-	-	-	<0.001
Female	11	31.5	8.4							-	-	-	-	-	-	-
All	27	31.4	10.0								-	-	-	-	<0.001	<0.001
Male	18	31.1	10.4	Jul							-	-	-	-	0.019	<0.001
Female	9	32.1	9.9								-	-	-	-	-	-
All	27	28.3	7.4									-	-	-	<0.001	<0.001
Male	16	28.9	8.3	Aug								-	-	-	-	<0.001
Female	11	27.3	6.1									-	-	-	0.017	-
All	27	30.7	9.3										-	-	<0.001	<0.001
Male	18	30.4	9.7	Sep									-	-	0.008	<0.001
Female	9	31.3	9.1										-	-	-	-
All	20	26.5	6.2											-	<0.001	<0.001
Male	12	26.2	7.0	Oct										-	-	<0.001
Female	8	26.9	5.0											-	-	-
All	27	34.4	10.0												-	<0.001
Male	18	34.5	11.2	Nov											-	<0.001
Female	9	34.1	7.6												-	-
All	14	37.6	13.3													-
Male	9	36.8	14.4	Dec												-
Female	5	39.0	12.7													-
All	17	44.2	19.8													
Male	11	47.6	23.4	Jan^†^												
Female	6	38.0	9.3													

All pairwise multiple comparison procedures (Holm-Šidák method) for differences of mean 25-OH-vitamin D serum concentrations (nmol/l) between months for all participants and separated between male and female participants with mean values ±SD and corresponding n per month,

^†^ denotes the January at the end of the overwintering

The equation of the fitted quadratic curve confirms the gradual drop in 25-OH-vitamin D over time and its progressive increase towards the end of the campaign (p<0.0001). Regarding dependency of 25-OH-vitamin D serum concentration on local daily sunlight-radiation at noon averaged over two-week intervals, we found a delay of the nadir of the fitted quadratic curve of the 25-OH-vitamin D serum concentrations compared to the nadir of a fitted quadratic curve of the local daily sunlight-radiation of 5.4 weeks. A linear regression analysis after compensation of this phase-shift through respective advancement of the 25-OH-vitamin D serum concentrations yielded Eq (b):

$$y=0.039x+29.48,\;r=0.396,\;r^{2}=0.156.$$ (b)

**Fig 4:**
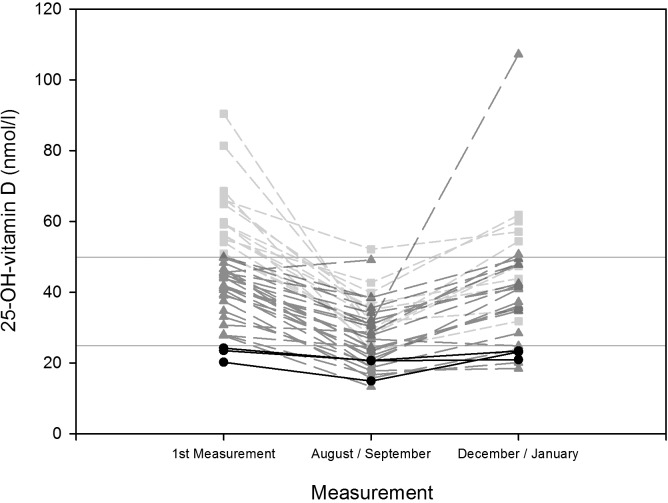
25-OH-vitamin D serum concentrations 2007–2012: Lineplot of individual values at first measurement vs. August and September vs. December and January, categorized for sufficient, insufficient and deficient values, for all participants. Changes of 25-OH-vitamin D serum concentrations from 2007 to 2012 based on individual values from the first measurement (n = 43), from August and September (n = 43) and from December and January (n = 31), categorized as sufficient (bright grey short dashed line and squares) insufficient (dark gray long dashed line and triangles) and deficient values (black solid line and circles), with additional horizontal lines denoting thresholds at 50 nmol/l and 25 nmol/l.

**Fig 5:**
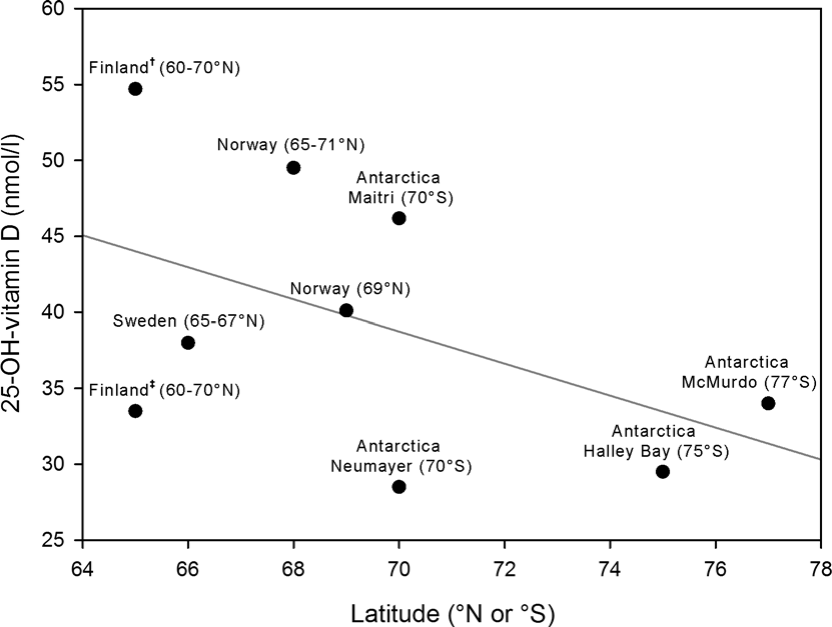
Scatterplot of mean 25-OH-vitamin D serum concentrations during local wintertime from Neumayer Stations 2007–2012 vs. other studies at high latitudes. Scatterplot for comparison of mean values of 25-OH-vitamin D serum concentrations during local wintertime found at Neumayer Stations 2007–2012 and other previous studies of comparable latitudes, for studies where a range of latitudes were given the mean latitude was used for this depiction, a linear regression curve has been added (grey line), ^†^ denotes results from study [61], ^‡^ denotes results from study [62].

The equation confirms the positive relationship between averaged local daily sunlight-radiation at noon and 25-OH-vitamin D serum concentrations measured 5.4 weeks later (p<0.001). Table 4 shows the results of the mean values of 25-OH-vitamin D per measurement month for all participants and separately for men and women. The percentage of participants with deficient values increased towards the period of complete darkness, while the percentage of participants with sufficient values decreased. For example in August none of the 27 subjects exhibited sufficient values, 66.7% showed insufficient ones and 33.3% deficient values. The percentage of deficient values decreased towards the end of the overwintering and the percentage of sufficient values increased, however, the percentages at the end of the overwintering did not reach the same values as at the beginning; in addition, none of the female subjects returned to sufficient values. As Fig 4 shows there was a considerable decrease in 25-OH-vitamin D serum concentrations from the first measurement to the period of complete darkness, however, the baseline values of 25-OH-vitamin D seemed to influence values later attained: all deficient values remained deficient values while insufficient values either remained insufficient or decreased to become deficient ones. Only values that were sufficient at the beginning remained so during the period of complete darkness (August-September). At the end of the overwintering (December-January) only values that had initially been sufficient, as well as two that had initially been insufficient, reached sufficient values. Several values that had initially been insufficient remained deficient towards the end (22.2%). Values that had initially been deficient remained so also at the end of the overwintering. Analysis of covariance revealed, that the covariate of baseline 25-OH-vitamin D serum concentration significantly affected the values of the dependent variable (25-OH-vitamin D serum concentration in August-September and December-January) (p<0.001). The other covariates did not significantly affect the 25-OH-vitamin D serum concentration in August-September and December-January: age (p = 0.068), gender (p = 0.688), baseline fat mass (p = 0.069) and station inhabitance (p = 0.066). Multiple linear regression models, in which the baseline 25-OH-vitamin D serum concentration was the only significant predictor for the 25-OH-vitamin D serum concentration during both subsequent periods (p<0.001), explained 33% (p = 0.015), and 42% (p = 0.022) respectively, of the variance of the 25-OH-vitamin D serum concentrations during August-September and December-January. With regard to the comparison to previous studies, Table 5 shows each study’s characteristics such as location, latitude, number of participants, mean values, and standard deviation based on their published results [10,46,60–65]. One-way ANOVA revealed that there was a significant difference between the study results (p<0.001). Multiple comparison analysis revealed that the results presented in this study during the period of complete darkness differed significantly from the mean values found by several other studies at comparable high latitudes during local wintertime. Fig 5 illustrates both the decrease in mean values of 25-OH-vitamin D found by the respective studies depending on latitude and the described low values found by this study in relation each other.

**Table 4:** 25-OH-vitamin D mean values per measurement month and percentages after categorization for sufficiency, insufficiency and deficiency.

	25-OH-vitamin D (nmol/l) mean ±SD per month											
	Jan	Mar	Apr	May	Jun	Jul	Aug	Sep	Oct	Nov	Dec	Jan^†^
All	48.4 ±15.0	39.0 ±10.8	45.9 ±4.1	33.8 ±10.4	31.6 ±7.6	31.4 ±9.8	28.3 ±7.3	30.7 ±9.2	26.6 ±6.0	34.4 ±9.8	37.6 ±12.8	44.2 ±19.2
n	31	43	4	27	27	27	27	27	20	27	14	17
Su %	35.5	18.6	25.0	3.7	0	3.7	0	3.7	0	3.7	0	23.5
In %	61.3	67.4	75.0	74.1	88.9	70.4	66.7	70.4	55.0	74.1	71.4	58.8
De %	3.2	14.0	0	22.2	11.1	25.9	33.3	25.9	45.0	22.2	28.6	17.7
Male	47.0 ±11.8	39.0 ±10.8	47.4 ±4.0	33.8 ±11.3	31.6 ±7.3	31.1 ±10.1	28.9 ±8.1	30.4 ±9.4	26.2 ±6.7	34.5 ±10.9	36.8 ±13.5	47.6 ±22.3
n	20	28	2	18	16	18	16	18	12	18	9	11
Su %	35.0	17.9	50.0	5.6	0	5.5	0	5.6	0	5.6	0	36.4
In %	60.0	71.4	50.0	72.2	87.5	66.7	68.8	72.2	58.3	72.2	66.7	45.4
De %	5.0	10.7	0	22.2	12.5	27.8	31.2	22.2	41.7	22.2	33.3	18.2
Female	51.0 ±19.2	38.8 ±10.8	44.4 ±3.6	33.7 ±8.5	31.5 ±8.0	32.1 ±9.3	27.3 ±5.8	31.3 ±8.5	26.9 ±4.7	34.1 ±7.2	39.0 ±11.4	38.0 ±8.5
n	11	15	2	9	11	9	11	9	8	9	5	6
Su %	36.4	20.0	0	0	0	0	0	0	0	0	0	0
In %	63.6	60.0	100.0	77.8	90.9	77.8	63.6	66.7	50.0	77.8	80.0	83.3
De %	0	20.0	0	22.2	9.1	22.2	36.4	33.3	50.0	22.2	20.0	16.7

25-OH-vitamin D serum concentrations mean values (nmol/l) per measurement month ±SD; percentage of given number of participants (n) per month after categorization of individual values as sufficient (Su: >50 nmol/l), insufficient (In: 25–50 nmol/l) and deficient (De: <25 nmol/l), for all participants and separately for both sexes,

^†^ denotes the January at the end of the overwintering

**Table 5:** Study characteristics and multiple comparison results regarding 25-OH-vitamin D serum concentrations from Neumayer Stations 2007–2012 vs. other studies of comparable high latitudes during local wintertime.

Study group	Location	Latitude (°N or °S)	Measurement Period	25-OH-vitamin D serum concentration (nmol/l mean ±SD)	n^†^	Multiple Comparison vs. Neumayer Stations p-value
Men prior fortification	Finland	60–70	January	33.5 ±9.2	49	0.229
Children prior fortification	Finland	60–70	September–April	54.7 ±17.0	50	<0.001
Adolescents with obstructive lung disease	Sweden	65–67	February–March	38.0 ±13.0	42	0.006
Middle-aged women	Norway	65–71	January–February	49.5 ±15.6	75	<0.001
Healthy male and female adults	Norway	69	February	40.1 ±14.6	41	<0.001
Healthy male expeditioners	Antarctica (Indian Maitri Station)	70	Midwinter	46.2 ±18.0	20	<0.001
Healthy male and female adults	Antarctica (German Neumayer Stations)	70	August–September	28.5 ±8.7	43	–
Healthy male expeditioners	Antarctica (British Halley Bay Station)	75	September	29.5 ±4.6	10	0.836
Healthy male expeditioners	Antarctica (US McMurdo Station)	77	August	34.0 ±12.0	7	0.548

Comparison of study groups, location, latitude, measurement period 25-OH-vitamin D serum concentration, number of participants and multiple comparison procedure (Holm-Šidák method) between results of respective studies and results of this study

^†^ denotes that n is reported for analyzed measurement period of local wintertime as stated in the respective study

## Discussion

Our study confirmes substantial decrements in 25-OH-vitamin D serum concentrations in overwinterers in Antarctica. Interestingly, 25-OH-vitamin D started to decrease in March, despite the fact that at this time of the year in Antarctica the ambient sunlight still prevails. As suggested by several authors [64,66], it is speculated that this decrease might be mostly due to indoor activities and protective clothing necessary for cold protection of the subjects, which blocks most of the UV-radiation. The changes of 25-OH-vitamin D serum concentrations over time during the years 2007–2012 follow a pattern that can be described by a quadratic trend. This substantiates previous findings regarding the seasonality of 25-OH-vitamin D metabolism and its dependence on UV-radiation [10,34,65]. The lowest values were attained during August and October, corresponding to a delay of the quadratic regression curve of 5.4 weeks after the nadir of local sunshine radiation (midwinter), which could be attributed to the half-life of 25-OH-vitamin D of up to three weeks [57], as well as the fact that foods consumed at the Neumayer Stations may have contained considerable amounts of 25-OH-vitamin D, as its supply through nutrition in general might have been underestimated, especially with regard to meat products [67–69]. After that time period, concentrations began to increase again but did not fully reach the pre-overwintering values, which is in concordance to previous findings in which vitamin D production in higher latitudes during the summer, was not sufficient to ensure adequate vitamin D serum concentrations throughout the year [70]. In contrast to several studies regarding overwintering in the Antarctic [71–73], we found a decrease in the BMI and FM towards the period of complete darkness. Several studies have shown an increase in 25-OH-vitamin D in subjects with a lower BMI or FM respectively [74,75]. However, our study shows a decrease in BMI, FM as well as 25-OH-vitamin D towards the period of complete darkness; therefore, the decline in BMI and FM does not seem to offset the decrease of 25-OH-vitamin D over time. Both men and women showed a similar decrease in 25-OH-vitamin D serum concentrations. This is in accordance with previous findings [15,76]. Furthermore, we observed no influence of age whereas other studies did find age-related differences [77–80]. This could be explained by the relatively young age of the participants in our study (the eldest being 60 years of age). Strikingly, no significant influence of residence in either station (Neumayer II or III) could be observed, which suggests similar conditions in both stations. This finding was rather unexpected given that Neumayer II was completely covered by ice, while Neumayer III is located above the surface. Most importantly, none of the subjects exhibited sufficient 25-OH-vitamin D serum concentrations during mid-winter (August) and only 3.7% did so during September. The percentage of participants exhibiting deficient values increased from 3.2% at the beginning of the overwintering to 33.3% in August, 25.9% in September and even 45% in October. The fact that the 25-OH-vitamin D serum concentrations at the end of the overwintering did not reach the same values as at the beginning could indicate that the overwintering has had a considerable impact on the overwinterers’ vitamin D metabolism. Less than 25% of all participants exhibited sufficient values at the end of the overwintering period. This, however, could also at least in part be attributed to the fact that not all participants gave blood samples for analysis during December and January at that time of the overwintering. Furthermore, it should be noted that a considerable proportion of the participants already started from low values at the beginning of the study: only 35.5% showed sufficient values then. Although we had applied rather conservative threshold values to categorize the measured 25-OH-vitamin D serum concentrations as sufficient, insufficient and deficient [39,40], we found high percentages for insufficient and deficient values throughout the overwintering and especially during the time of complete darkness. The analysis of covariance and multiple linear regression analysis revealed that the baseline values of 25-OH-vitamin D had significant influence on the values subsequently developed during the periods of complete darkness. From several independent parameters, only the 25-OH-vitamin D serum concentration taken at the first measurement significantly affected subsequent concentrations as a covariant during August and September and at the end of the overwintering during December and January. This parameter was the only one to significantly predict the subsequent values during these periods. This is in accordance with a previous study that concluded that summer 25-OH-vitamin D serum concentrations influence the concentrations during the following winter in individuals living in Denmark [34]. Finally, as was shown in the statistical analysis, the mean results of 25-OH-vitamin serum concentrations of overwinterers at the Neumayer Stations during periods of complete darkness presented in this study were significantly lower than those found by several other previous studies of comparable high latitudes conducted during local wintertime. Only the findings from two Antarctic overwintering studies conducted in research stations further south during local wintertime [10,60] and one study conducted in Finland during local wintertime [62] yielded mean results that did not differ significantly from the results from the Neumayer Stations presented in this study. It is conceivable that operational demands and circumstances inherent to the study setting, such as protective clothing, limited food (little fresh fruit and vegetables, high amounts of industrially processed foods) [45,48,81] as well as confined space [82], psychosocial isolation [83], reduced physical activity [84] with consequences for vitamin D serum concentrations [85], might have led to these low mean values during the period of complete darkness. It is conceivable that the combination of adverse environmental conditions in isolated and confined spaces, such as remote Antarctic research stations, may lead to a complex deterioration of several physiological systems like metabolism, vitamin D and hormone balance, circadian rhythm, sleep and mood changes as well as energy intake and expenditure. It could be argued that these mechanisms are challenged in such environments, combining the effect through complex interactions, as it has been previously shown [86,87]. To strengthen the findings of this study, future studies in this setting could include other outcome measures such as changes in parathyroid hormone concentrations. Further research seems warranted to investigate interactions in isolated environments in high latitudes. Our findings give rise to the recommendation, that at remote research stations, such as Neumayer, vitamin D supplementation should be considered [60] with doses higher than previously thought [88] or the diet should be composed of foods naturally rich in vitamin D [47,89]. These findings might be extended to other environments of similar conditions for which Antarctic residence is an analogue such as long duration space missions [90].

We conclude that insufficient UV-radiation and low baseline 25-OH-vitamin D serum concentrations, as well as a complex set of conditions inherent to the overwintering in an isolated and confined research station–like psychosocial isolation, altered circadian rhythm and change in nutrition, energy intake and expenditure–might have contributed to these results. Further studies are warranted to evaluate the complex interaction of these parameters, i.e. for example to investigate how artificial UV-light sources inside the station might induce 25-OH-vitamin D production as well as the impact of controlled vitamin D supplementation in counteracting vitamin D deficiency and thus to enhance the physical and psychological well-being of overwinterers in Antarctica as well as astronauts in space.

## Abbreviations (in alphabetical order)

7-DHC: 7-dehydrocholesterol
ANCOVA: Analysis of covariance
ANOVA: Analysis of variance
Apr: April
Aug: August
BIA: Bio impedance analysis
BMI: Body mass index
BodPod: Whole body plethysmography
De: Deficient
Dec: December
Feb: February
FM: Fat mass
In: Insufficient
Jan: January
Jul: July
Jun: June
Mar: March
N: North
Nov: November
Oct: October
RM: Repeated measures
S: South
SD: Standard deviation
Sep: September
Su: Sufficient
UV: Ultraviolet
W: West
